# Improving the Proteome-Mining of *Schizophyllum commune* to Enhance Medicinal Mushroom Applications

**DOI:** 10.3390/jof11020120

**Published:** 2025-02-05

**Authors:** Anthea Desiderio, Lorenzo Goppa, Carlo Santambrogio, Stefania Brocca, Simone Buratti, Carolina Elena Girometta, Meghma Sarkar, Maria Teresa Venuti, Elena Savino, Paola Rossi, Emanuele Ferrari

**Affiliations:** 1 Department of Earth and Environmental Sciences (DSTA), University of Pavia, 27100 Pavia, Italy; anthea.desiderio01@universitadipavia.it (A.D.); lorenzo.goppa01@universitadipavia.it (L.G.); simone.buratti@unipv.it (S.B.); carolinaelena.girometta@unipv.it (C.E.G.); elena.savino@unipv.it (E.S.); 2 Department of Biotechnology and Biosciences, University of Milano-Bicocca, 20126 Milan, Italy; carlo.santambrogio@unimib.it (C.S.); stefania.brocca@unimib.it (S.B.); 3 Department of Biology and Biotechnology “L. Spallanzani”, University of Pavia, 27100 Pavia, Italy; meghma.sarkar01@universitadipavia.it (M.S.); mariateresa.venuti01@universitadipavia.it (M.T.V.); 4 Molecular Ecology Group (MEG), Water Research Institute (CNR-IRSA), National Research Council of Italy, 28922 Verbania, Italy

**Keywords:** protein isolation, proteomics, extraction method, medicinal mushroom, mycelium

## Abstract

This study presents the first comprehensive proteomic profile of an Italian strain of *Schizophyllum commune*, a highly heterogeneous white-rot fungal species with significant potential for industrial, nutraceutical, cosmeceutical, and clinical applications. Three protein extraction methods and their impact on yield and resulting protein composition have been compared. Results revealed that the combination of Tris–Cl and urea increases the total protein yield and the variety of enzymatic species related to pivotal pathways. Notably, over 2000 proteins were identified, including enzymes involved in the growth and development of mycelium, trehalose biosynthesis, and different types of carbohydrate-active enzymes (CAZymes). These enzymes are crucial for nutraceutical and agro-industrial applications of *S. commune*. The multiple-step proteomic approach used could be a model for investigating other fungal species.

## 1. Introduction

Medicinal mushrooms (MMs) have been used for centuries for their therapeutic and nutritional benefits, thanks to their bioactive compounds like polysaccharides, terpenoids, and proteins [1,2]. Known for their immunomodulatory, anticancer, antiviral, and anti-inflammatory properties, they are a key research focus [3]. Among the MMs, *Schizophyllum commune* Fr. is a widespread saprotrophic basidiomycete found on broadleaf trees. It has diverse biological effects, including anti-inflammatory, antimicrobial, antioxidant, and anticancer properties [4]. A hallmark molecule of this mushroom is the schizophyllan (SPG), a β-(1→3)/(1→6)-glucan extracellular polysaccharide [5]. Schizophyllan is valued for its immunomodulatory, antitumor, and potential wound-healing properties, acting as a biological response modifier [6] and exhibiting antioxidant effects [7]. *S. commune* produces bioactive compounds, including polyphenols, which have antioxidant and anti-inflammatory effects by neutralizing free radicals and reducing oxidative stress [8].

As a lignicolous fungus, *S. commune* plays a vital role in nutrient cycling by decomposing lignocellulosic materials through a variety of hydrolytic enzymes, including cellulases, endoglucanases, β-glucosidases, and hemicellulases such as xylanases [9,10]. The wood-degrading ability of *S. commune* could be crucial for industrial applications such as bioremediation and biofuel production [11,12]. Oxidoreductases, including laccases and various peroxidases, are responsible for their roles in lignin degradation, dye decolorization, and synthesis of valuable aromatic compounds [13]. Under laboratory conditions, *S. commune* easily grows on various media, forming fluffy-white mycelia. It is used as a model organism in functional genomics [14] and for applications in mycomaterials [15]. Despite these potential applications, the protein profile of *S. commune* remains partially unknown.

A critical part preceding fungal proteome analysis is the extraction process. Efficient protein extraction methods are essential for mushrooms due to their complex tissue structure and robust cell walls composed of chitin, glucans, and proteins. Common approaches include mechanical disruption, such as grinding tissues in liquid nitrogen or using bead-beating techniques to break the cell walls and release intracellular contents. Chemical treatments with detergents (e.g., SDS, Triton X-100), chaotropic agents (e.g., urea, thiourea), and various solvents (e.g., ethanol, methanol, acetone) are often employed to solubilize proteins. Additionally, protease inhibitors are used to minimize protein degradation during the extraction process. These techniques, tailored to the unique properties of mushrooms, provide the foundation for robust proteomic analyses. While efficient protein extraction is critical due to the complexity and robustness of mushroom tissues, the indiscriminate use of a full range of chemical and mechanical approaches should be avoided. Instead, extraction methods should be tailored to the specific objective, avoiding unnecessary steps and minimizing the use of solvents and reagents. This approach aims to combine both simplicity and efficacy in the extraction process [16].

Currently, integrating data from proteomics, transcriptomics, and metabolomics provides insights into enzyme families, regulatory networks, and specific molecular pathways. Recent advances in proteomics have enabled the detailed analysis of fungal metabolism, supported by complete genomes and extensive genomic data. Early research primarily focused on human pathogens, especially filamentous ascomycetes, in which specific proteins play key roles in host recognition and virulence [17,18]. Proteomics studies on MMs began around the 2000s, mainly on *Ganoderma lucidum* (Fr.) P. Karst., driven by a growing interest in their biological properties and potential biotechnological use [19]. Proteomics has also been successfully applied to cultivable fungi and wood decayers such as the model species *Phanerochaete chrysosporium* Burds. The focus of these studies was to understand the enzyme patterns involved in the degradation of lignocellulose and persistent organic pollutants [20,21]. This highlights the potential of proteomics to reveal insights beyond transcriptomics, particularly under varying growth conditions that significantly influence enzymatic networks like those involved in lignocellulose degradation [22,23]. Proteomics is essential for characterizing protein expression under specific conditions, aiding in the selection of fungal strains with desirable biotechnological traits. However, proteomic profiles are highly sensitive to experimental variables, including culture conditions and protein extraction methods [24,25].

A few studies on the *S. commune* protein profile, under peculiar environmental conditions, have focused on specific aspects such as secreted proteins related to wood degradation [26] and protein expression profiles in response to the mycoparasite *Trichoderma viride* Pers. [27], and various metabolic pathways for metal tolerance [28]. *Leucaena leucocephala* wood (LLW) was used as a substrate for *S. commune* ISTL04 to produce extracellular proteins and schizophyllan under submerged fermentation [26]. Proteome analysis involved SDS-PAGE separation and nano LC-MS/MS profiling, identifying 79 extracellular proteins classified by their biological roles. Maximum production was observed on day 18, yielding 8.53 mg mL^−1^ of sugar, 391 mg L^−1^ of extracellular protein, and 4.2 g L^−1^ (0.21 g g^−1^ LLW) of exopolysaccharide (EPS). EPS, identified as schizophyllan, was characterized using FTIR and GC-MS. Another study investigated protein profile changes in *S. commune* when paired with the biocontrol fungus *Trichoderma viride* for 48 h [27]. Using two-dimensional (2D) protein gel electrophoresis and matrix-assisted laser desorption/ionization time-of-flight mass spectrometry, they revealed significant proteomic changes. *S. commune* showed a 61% increase in proteins related to transcription and translation, along with a 17% rise in proteins for cell walls and hyphal biogenesis, while metabolism-related proteins decreased by 64%. Meanwhile, *T. viride* exhibited mycoparasitic behavior, with elevated levels of proteins linked to proteolysis and carbohydrate metabolism. These findings highlight the *S. commune* upregulation of mechanisms to counteract *T. viride* mycoparasitic activity, particularly cell wall lysis and antibiosis. This study provides insights into natural resistance mechanisms and has implications for the design of improved biocontrol strategies. Finally, the investigation of inositol phosphate signaling in *S. commune* highlighted its role in growth, sexual reproduction, and metabolic adaptation [28]. Overexpression of the corresponding gene revealed its involvement in cell wall integrity, intracellular trafficking, and significant impacts on mushroom formation and metabolism, as evidenced by proteomic analyses. Altered inositol signaling also enhances metal tolerance (e.g., to cadmium, cesium, and zinc), with metal exposure reducing intracellular calcium levels and influencing kinase and phosphatase expression within the inositol cycle.

These examples of research demonstrate considerable diversity, especially in the method of extracting and identifying proteomic profiles. In this study, the proteomic profile of *S. commune* mycelium was investigated to identify functional metabolic pathways with potential applications in agro-waste recycling, industrial production, and human or animal health. The culture medium (malt extract) and extraction procedures were chosen to minimize the loss of proteins possibly involved in crucial biological pathways. We suggest that the multiple-step proteomic approach described, integrating different databases, could be used as a model for investigating other fungal species.

## 2. Materials and Methods

### 2.1. Fungal Strain Isolation, Identification, and Conservation

The basidiome of *S. commune* was collected from *Populus alba L*. in Bereguardo (Pavia, Italy) and isolated in pure culture in Petri dishes containing 2% *w*/*v* Malt Extract Agar (MEA). The strain was identified by macro- and micro-morphological characteristics followed by molecular analysis. Total genomic DNA from lyophilized mycelium was extracted using the Nucleospin Plant II kit (Macherey-Nagel, Düren, Germany). The primer pair ITS1-ITS4 was used to amplify the Internal Transcribed Spacer (ITS) region by Polymerase Chain Reaction (PCR) as reported in the method [29]. The fungal strain in pure culture is cataloged as MicUNIPV_S.c.1 in the Fungal Research Culture Collection (MicUNIPV) of the Mycology Laboratory at the Department of Earth and Environmental Sciences (DSTA) (University of Pavia, Italy). The strain is maintained both at 4 °C in MEA and −80 °C in a water-glycerol solution for long-term conservation [30].

### 2.2. Mycelium Growth

*S. commune* mycelium was grown in MEA Petri dishes for 15 days at 25 °C in the dark. From the fully colonized plate, 10 portions of colonized medium (surface about 0.25 cm^2^ each) were sterilely withdrawn and inoculated into flasks (capacity of 1 L) containing 400 mL of 2% *w*/*v* ME. Before inoculation, flasks were sterilized by autoclave (121 °C, 20 min) and sealed with raw cotton to facilitate gaseous exchange. Incubation was carried out in dark and static conditions at 25 °C. After 15 days, mycelium was washed with sterile deionized water, lyophilized for 24 h at −50 °C and 1 mbar and finally stored at −20 °C.

### 2.3. Protein Extraction Procedures

The extraction procedures were developed based on a previous study [31], combining mechanical disruption using a bead mill with extraction of both soluble and insoluble protein fractions using different buffers. Lyophilized mycelium was frozen in liquid nitrogen for 60 s and then finely grounded using a pestle and mortar. The resulting powder was subjected to three distinct extraction methods (A1, A2 and B).

Method A1: 100 mg of fungal powder was suspended in 1 mL of 10 mM Tris–Cl buffer pH 8 and homogenized with 100 mg of glass beads (diameter mechanical ~500 µm), using a FastPrep-24 (MP Biomedicals, Irvine, CA, USA for 4 cycles of 30 s at 6.5 m/s speed). The sample was then centrifuged (15 min, 14,000× *g*, room temperature) to separate the soluble and insoluble fractions. The soluble fraction was named sample A1.

Method A2: The insoluble fraction obtained with Method A1 was washed twice with acetone, resuspended in a solubilization buffer (8 M urea in 10 mM Tris–Cl buffer, pH 8) and incubated at room temperature for 24 h before brief centrifugation was applied to remove particulate matter and glass beads. This fraction was named sample A2.

Method B: 100 mg of the fungal powder was suspended in 1 mL of 10% (*v*/*v*) trichloroacetic acid (TCA) for protein precipitation. The sample was homogenized as previously described (FastPrep-24—MP Biomedicals, Irvine, CA, USA—4 cycles of 30 s at 6.5 m/s speed, in the presence of glass beads) and centrifuged (15 min, 14,000× *g*, room temperature). The resulting pellet was washed twice with acetone, resuspended in a solubilization buffer (8 M urea in 10 mM Tris–Cl buffer, pH 8) and incubated at room temperature for 24 h. After incubation, the sample was briefly centrifuged to remove particulate matter and glass beads.

Briefly, the use of three distinct extraction methods resulted in the generation of three types of samples: A1 (soluble fraction from Method A1), A2 (insoluble fraction from Method A1), and B (pellet from Method B). The protein content of each sample was quantified by absorbance spectroscopy employing a Bradford assay (with absorbance calibrated against a bovine serum albumin standard). Protein composition was assessed by loading 10 μL of each sample on a 14% SDS-PAGE gel, run on a mini-gel apparatus (Bio-Rad, Berkeley, CA, USA), according to the Laemmli method [32].

### 2.4. LC-MS/MS Analysis

Samples A1, A2, and B were diluted to a final concentration of 10 mM Tris–Cl buffer (pH 8) and 4 M urea. The extracted proteins were reduced with 10 mM dithiothreitol, alkylated with 50 mM iodacetamide and digested with trypsin 16 h at 37 °C (with urea diluted to a final concentration of 2 M). The resulting peptides were desalted using Ziptip C-18 columns, lyophilized and suspended in 0.1% formic acid. The samples were then analyzed by LC/MS using an Orbitrap Fusion instrument (Thermo Fisher Scientific, Waltham, MA, USA) equipped with an HPLC system (EASY-1000). Peptides were separated on a 50 cm C-18 column (Thermo Fisher Scientific, Waltham, MA, USA) using a 120 min gradient (solvent A: 100% milliQ water, 0.1% formic acid; solvent B: 20% milliQ water, 80% acetonitrile, 0.1% formic acid). Detection was performed in the orbitrap analyzer, and fragmentation occurred in the ion trap analyzer using high-energy collision dissociation (HCD) with helium gas. Two technical replicates were performed for each sample.

### 2.5. Protein Identification, Functional and Statistical Analysis

Protein identification was performed with MaxQuant 2.0.3.0 [33] against the *S. commune* database downloaded from Uniprot in April 2023. All parameters were set at default values except for the following. Mass search tolerances were set to 10 ppm for MS and 0.6 Dalton for MS/MS. Carbamidomethylation of cysteines was set as a fixed modification, while methionine oxidation was set as a variable modification. The possible missed cleavages for trypsin/P were set to 3. The false discovery rate was set to 1% at peptide and protein levels based on a target/decoy search. Unique and razor peptides were used by Peptide Spectrum Match (PSM) to quantify proteins. For each individual technical replicate, the extracted proteins, taking into account the average spectral count among the three replicates, were quantified.

The mass spectrometry proteomics data have been deposited to the ProteomeXchange Consortium via the PRIDE partner repository [34] with the dataset identifier PXD048036 and 10.6019/PXD048036.

Perseus was used for post-processing data and statistical analysis. The sample comparison of the enriched Cellular Component terms was performed with FunRich using the Uniprot Database [35]. DAVID was used for functional analysis and for statistical analysis of the enrichment of Gene Ontology terms, and a *p*-value < 0.01 was considered significant [36]. The enriched terms were then clustered according to semantic similarity using Revigo [37].

## 3. Results

### 3.1. Different Protein Content and Proteomic Identification in the Three Extracts

The three samples resulting from the extraction procedures (A1, A2, and B) contained different total protein amounts according to Bradford assay and SDS-PAGE analysis (Figure S1). Specifically, sample A1 corresponds to soluble proteins extracted under native conditions using an aqueous buffer containing 10 mM Tris–Cl at pH 8, while sample A2 represents the insoluble proteins that were subsequently extracted with urea in the same buffer. Sample B, on the other hand, consists of proteins precipitated with trichloroacetic acid (TCA) and then solubilized in urea, representing a different extraction approach targeting both soluble and insoluble fractions.

Sample A1 contains about four times the total protein content (3.1 ± 0.1 mg/mL) compared to A2 (0.81 ± 0.08 mg/mL) and 10 times that of sample B (0.35 ± 0.05 mg/mL). Interestingly, the number of different proteins identified through the proteomic approach did not correlate with the total protein content measured by the Bradford assay. This means that, despite its higher protein content, sample A1 exhibits lower protein diversity compared to the other samples. Such a discrepancy is unlikely to stem from an artifact in the protein concentration measurement arising from the diverse detectability of specific proteins owing to their composition or size. The Bradford assay applied to complex protein mixtures is generally robust and less prone to significant biases of this nature.

**Figure 1:**
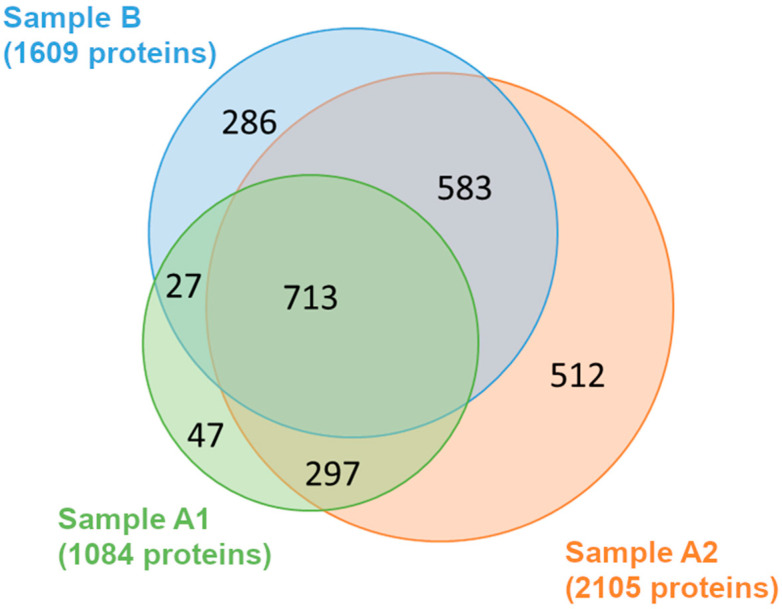
Venn’s diagram shows the number of identified extracted proteins in samples A1, A2, and B. Around 30% of the proteins were identified in all samples (713 proteins), indicating a core of proteins easily extracted with all three methods. Sample A2 showed the highest number of unique proteins, which were not identified in the other samples (512 proteins).

As shown in the Venn diagram (Figure 1), a total of 2465 proteins were identified in the three samples (File S2). Around 30% of these proteins (713) were common in all three samples, confirming the presence of a protein core that was extracted independently from the extraction procedure (Figure 1).

Sample A1 yielded the lowest number of identified proteins (n = 1084). Of these, 297 proteins were only shared with sample A2, 27 with sample B, and 47 were unique to A1. Sample A2 had the highest number of identified proteins (n = 2105). Apart from those shared with A1, A2 also had a significant overlap with sample B (n = 583) and 512 proteins unique to it. Sample B yielded a number of n = 1609 identified proteins, of which 286 proteins are unique for this sample (Figure 1).

### 3.2. Cellular Compartmentalization of the Extracted Proteins: The Gene Ontology Cellular Component Analysis

Enrichment analysis based on the Gene Ontology Cellular Component (GO-CC) terms was assessed to identify the cellular compartment of the proteins identified, comparing with the list of proteins in the *S. commune* reference proteome database. GO-CC terms were found for 906 out of the total 2465 proteins identified (about 37%). Considering each sample separately, the number of proteins to which a GO-CC term was assigned was 394 for sample A1 (~37%), 815 for sample A2 (~38%), and 562 for sample B (~35%).

In particular, in 12 cellular components, at least one extract showed a statistically significant enrichment compared to the whole proteome of *S. commune* (Table 1). Notably, cytoplasm and ribosomal proteins were enriched in all three samples, but a higher number of proteins were found in extract A2. The three methods clearly differ in extraction power, with sample A2 showing the highest enrichment of the 12 CC terms selected (Table 1). The analysis of GO-CC was focused on the differences among the three extracts (Table 1).

**Figure 2:**
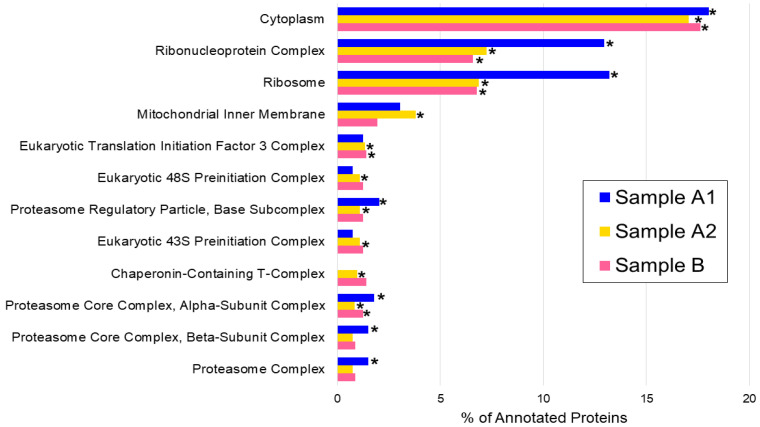
Percentage of annotated proteins for each GO-CC term. For each sample, the percentage has been calculated as the ratio between the number of proteins annotated for GO-CC terms and the total number of annotated proteins in the sample (394 for sample A1, 815 for sample A2, and 562 for sample B). The significant enrichment in the samples with respect to the whole proteome (as reported in Table 1) is indicated by the symbol * beside the bars (*p*-value < 0.01).

The percentage of annotated proteins for each GO-CC term is calculated on the ratio between the number of proteins annotated for that GO-CC term and the total number of annotated proteins in the sample (394 for sample A1, 815 for sample A2, and 562 for sample B, Figure 2).

To identify in detail the differences in protein abundances as detected by CCs in all three samples, the Peptide Spectrum Matches (PSMs) were assessed. PSMs (or spectral count) allow the comparison of protein abundances without using any labeling in the preparation of samples and are based on the number of peptides identified for each protein group. When comparing sample A2 with sample A1, an increase in the abundance of membrane proteins, including mitochondrial, Golgi apparatus, and endoplasmic reticulum membranes, was observed (Table 2). In agreement with the biophysical and chemical characterization of membrane proteins, a huge number of proteins were insoluble in aqueous solutions and thus extracted by urea.

The same pattern was observed when comparing samples A2 and B (Table 3). The proteins associated with respirasome, ribosome, and various organelle membranes (Endoplasmic Reticulum, Golgi, and Mitochondria) showed the highest fold change values, meaning that the proteins from these compartments were more expressed in sample A2. Consequently, it appears that the method used for sample A2 is the most effective for isolating proteins from membranes among the different extraction methods under investigation.

### 3.3. Functional Analysis and Characterization of the Extracted Proteins

**Figure 3:**
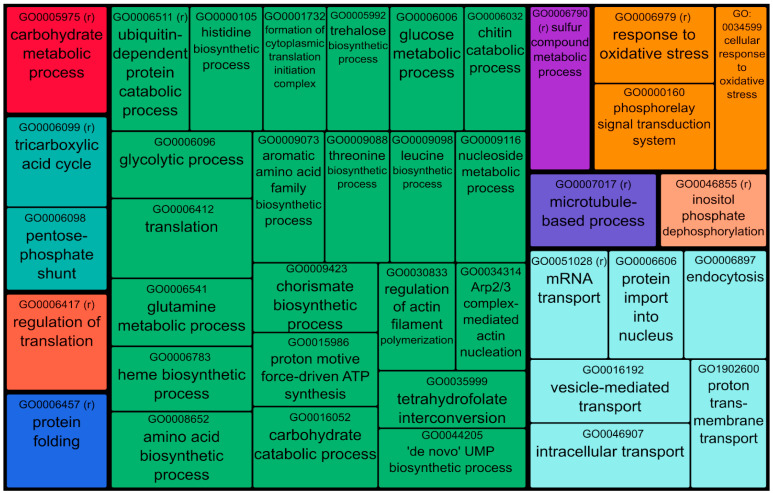
Treemap view clustered with REVIGO of GO Biological Process terms associated with fungal proteins. Each rectangle is a single GO term or a representative term of a cluster of semantically similar GO terms (indicated by r in brackets) with size related to the fold enrichment with respect to the whole proteome. The “superclusters” of related terms are visualized with different colors.

A functional analysis was performed to understand which metabolic pathways are present in the three extracts. Proteins identified in the three samples (2465 proteins) were pooled together and processed with DAVID https://david.ncifcrf.gov/ (accessed on 3 February 2025) [36]. Considering all samples, 47 Gene Ontology Biological Process terms were found to be enriched (File S3). Consequently, the software Revigo was employed to remove redundant GO terms, cluster them by similarity, and visualize data in a treemap. Enriched terms could be classified into 10 superclusters indicated by different colors (Figure 3). Each supercluster is composed of semantically and functionally related GO-BP terms or clusters of semantically similar GO-BP terms. These are represented by rectangles whose size is proportional to the fold enrichment in protein expression with respect to the whole proteome. Fold enrichment is the ratio quantifying how much more (or less) a specific category is represented in a protein subset compared to what would be expected by chance in the entire dataset. This metric highlights biological processes, molecular functions, or cellular components that are statistically overrepresented and thus relevant to the biological context of the analyzed subset, aiding in the interpretation of proteomics data.

The treemap exhibits that the green supercluster is the one that comprises more GO-BP terms related to the biosynthesis and degradation of proteins and polysaccharides. It is important to note the presence of clusters of trehalose biosynthesis (GO:0005992) and carbohydrate catabolic processes (GO0016052) within the green supercluster. Trehalose production plays a vital role in fungal metabolism, supporting the growth and development of mycelium while also acting as a thermo-protector. Carbohydrate catabolic processes involve a range of enzymes responsible for the biosynthesis, modification, binding, and breakdown of carbohydrates, collectively known as carbohydrate-active enzymes (CAZymes or CAZy) [38]. Furthermore, considering the GO-BP terms, an enrichment of proteins involved in intra- and extracellular transport (light blue supercluster) and in response to oxidative stress (orange supercluster) were observed too.

The light sea green supercluster consists of two important metabolic processes: the pentose-phosphate shunt and the tricarboxylic acid cycle. It is important to emphasize the significance of these pathways, with a particular focus on the tricarboxylic acid cycle, which is linked to the growth and development of mycelium. The key enzymes involved in this metabolic pathway, isocitrate lyase and malate synthase, are part of the glyoxylate cycle enzyme. This cycle appears to be involved in the transition phase between vegetative growth and the development of the fruiting body [39,40]. Across the other superclusters, a wide range of activities were observed, including metabolic processes, protein folding, cell division, and gene control processes, protein folding, cell division, and gene control.

Among the biological processes enriched for *S. commune*, the attention was then focused on the metabolic pathways that are promising for industrial and health applications (Table 4). The most interesting molecular pathways considered were trehalose biosynthesis, response to oxidative stress, pentose-phosphate shunt, tricarboxylic acid cycle, CAZymes and inositol phosphatase dephosphorylation. A quantitative comparison (based on PSMs) of the expression of enzymes related to these processes in the three samples was performed. In each metabolic pathway, specific enzymes known as “key enzymes’‘ (indicated in bold and underlined) have been identified based on literature data. These enzymes are of significant scientific interest due to the crucial role they play in the pathway and their potential applications in various fields. Significantly higher amounts of most proteins, particularly key enzymes, were observed in sample A2 across all the metabolic pathways under investigation.

As shown in the table, sample A2 appears to be the richest not only in the quantity of extracted proteins but also in the variety of enzymatic species related to pivotal pathways. In particular, some enzymes that are present in Table 4 will be described for their application in the discussion. This section may be divided by subheadings. It should provide a concise and precise description of the experimental results, their interpretation, and the experimental conclusions that can be drawn.

## 4. Discussion

This study represents the first proteomic analysis of an Italian strain of *S. commune*, a species that shows remarkable morpho-physiological and molecular variability. It is expected that traits specific to this population can be identified using proteomic analysis [41,42].

Mycelium grown in a nutrient-rich medium (ME 2% *w*/*v*) resulted in the identification of over 2000 proteins involved in different metabolic pathways.

In proteomic analyses, the extraction procedure is a crucial step in obtaining reliable and reproducible results since it significantly influences both the yield and diversity of the proteins obtained. In this study, to minimize protein loss, the mycelium of *S. commune* was first subjected to bead milling, a mechanical lysis method for protein extraction generally recognized as non-selective [43]. The following extraction was carried out under different conditions: (i) native conditions, separating the soluble fraction (sample A1) and the insoluble fraction extracted with urea (sample A2), or (ii) in the presence of TCA (sample B). Each sample differs not only in protein extraction yield but also in the diversity of the identified proteins.

In particular, sample A2, representing the insoluble fraction subsequently extracted with urea and Tris-Cl, showed the highest number of identified proteins (2105), with a significant number of unique proteins not found in the other samples. Sample A2 had the highest number of proteins, indicating that urea was a more effective agent for protein extraction.

Our results are consistent with those of other studies. Taunk et al. [44] showed that the combination of urea and Tris–Cl maximizes the yield of protein extraction in mammalian cells. Chi and Cho [45] showed that in soybean meal samples, the use of urea increased protein extraction efficiency by 90%.

Furthermore, TCA (used for sample B), traditionally used for protein isolation, resulted in an overall decrease in the protein yield. Even with the inclusion of appropriate detergents, TCA was found to be less effective for total protein extraction compared to other reagents [46,47].

It should be noted that the choice of extraction method affects not only the total yield but also the proteins that can be recovered. In particular, sample A2 showed enrichment of proteins from various cellular compartments, including the cytoplasm, membranes, and ribosomes, highlighting the importance of selecting the most effective method for isolating proteins from complex samples. Notably, no enzymatic treatments were used to digest the cell wall to keep the extraction process as simple and reproducible as possible.

Relatively few transcriptomic and proteomic analyses on *S. commune* have been performed so far [26,27,28,48]. In contrast to a previous study that evaluated the degradative activity of *S. commune* ISTL04 through the analysis of extracellular enzyme expression [26], our study focuses on characterizing the intracellular proteome of *S. commune* under standard growth conditions without external stimuli that could influence enzyme expression. Other proteomics studies focused on the relationship between *S. commune* and the antagonist *Trichoderma viride*, investigating their defense mechanisms [27] and on a single metabolic pathway, the inositol phosphate, and its impact on fungal metabolism [28]. In our work, the broad range of extracted proteins allows for the investigation of multiple metabolic pathways by using widely employed databases, such as Gene Ontology and Uniprot. Consequently, our experimental design is more easily replicable. Since the fungus is not induced to produce specific enzymes or activate specific metabolic pathways, the extracted proteins are more abundant in both total number and diversity.

Several proteins enriched in metabolic pathways under the growth conditions employed were examined in depth. First, attention was placed on the trehalose biosynthesis pathway [49] since this carbohydrate is known as a food stabilizer and protective agent against oxidative stress, holding substantial value in the industry [50,51]. In our samples of *S. commune*, the following key enzymes have been successfully identified: trehalose synthase (UniProt accession number D8QC44), trehalase (D8QIA1), and trehalose phosphorylase (Q2HZZ3) (see Table 4). It is worth noticing that only method A2 allowed for the extraction of these enzymes in sufficient quantities to enable their subsequent identification. This underscores the importance of using a reliable extraction procedure to avoid the loss of important biological information. Trehalose is known for its effectiveness as a food stabilizer and a protective agent against oxidative stress. It holds substantial value in the industry, with applications extending to the pharmaceutical and cosmetic sectors due to its thermal-protective properties [52,53]. The expression of enzymes involved in the trehalose biosynthesis pathway by *S. commune* shows potential for the development of the enzymatic synthesis of trehalose with significant cost savings and reduced production time compared to the expensive and complex chemical synthesis currently in use.

The present proteomic analysis also detected 2 out of 6 major classes in the family of CAZy family carbohydrate-active enzymes [54,55]: glycosyl hydrolases (GHs) and glycosyl transferases (GTs). On the other hand, polysaccharide lyases (PLs), carbohydrate esterases (CEs), carbohydrate-binding modules (CBMs) and auxiliary activities (AAs) did not exceed the detection limit. In mushrooms, CAZymes play vital roles in the development of fruiting bodies [56], in cell wall formation, and in nutrient uptake, as well as in the breakdown of lignocellulosic substrates [40]. Researching CAZymes can enhance our understanding of biological processes and improve fungal cultivation [51]. CAZymes are crucial for breaking down complex carbohydrates, aiding digestion, and promoting nutrient absorption in the human gut microbiota, where the human genome encodes only 17 such enzymes. Specifically, glycoside hydrolase (GH) and glycosyl transferase (T) enzymes are vital for a healthy microbiome [57,58,59]. CAZymes also play a role in immune defense and reducing inflammation, with decreased expression linked to various diseases. Therefore, we aimed to investigate which CAZymes are expressed by *S. commune* under specific growth conditions.

Among the various GH enzymes, alpha-mannosidase (D8QGJ4) was found in higher amounts in the extract A2. This CAZyme has been identified as a potential target for tumor and cancer treatment [60] and is involved in the post-translational modification of secreted proteins [61]. Other GH enzymes significantly expressed in *S. commune* are 1,4-alpha-glucan (D8PS34) and trehalose phosphorylase (Q2HZZ3), both of which are implicated in the biosynthesis of Trehalose. Also, in this case, sample A2 showed a higher presence of these two enzymes, which play a crucial role in the food industries [62]. In *Hypsizygus marmoreus* (Peck) H.E. Bigelow, the expression of 1,4-alpha-glucan and trehalose phosphorylase was observed during the primordium phase of growth and not during the mycelia growth [63]. In contrast, the expression of these enzymes was high as early as the mycelial growth stage of the *S. commune* used in this study.

Finally, beta-glucosidase enzymes (D8PJV0, D8PU51, D8PUK9, D8Q8Q6) are relevant due to their hydrolytic action in degrading lignocellulosic components, thereby facilitating the growth and subsequent development of mushrooms [63,64]. Recently, β-glucosidase has gained attention in the industrial sector as a valuable enzyme for repurposing food waste [65]. Hence, it can be deduced that extract A2 contains the highest amount of all the CAZymes previously discussed.

Among the CAZy family, two enzymes belonging to the GTs class were identified: α-galactosidase (D8PQ92) and β-galactosidase (D8Q3Q9, D8PK76, D8PM67, and D8Q3Q9). Alpha-galactosidase can break down galactose residues found in oligosaccharides, such as raffinose-family oligosaccharides. Nowadays, it is used to reduce stachyose and raffinose in soybean flour for industrial applications in the food and feed industries [66]. However, these enzymes could be applied in agriculture, food production, and biomedicine [67,68].

Beta-galactosidases derived from fungi find innovative applications in the production of prebiotic compounds, recycling agro-industrial wastes and removing lactose from dairy products [69,70,71,72]. Based on promising preliminary results, mushroom-derived mixtures containing β-galactosidases may offer more economical solutions for bioethanol production from cheese whey [23].

Among the 10 superclusters presented in the treemap, significant enrichment was also observed in the pentose-phosphate shunt. This metabolic pathway is crucial for the biosynthesis of erythritol [73]. This molecule influences not only mycelial growth but also the formation of the fruiting body [74]. The main enzymes involved in this metabolic pathway are 6-phosphogluconate dehydrogenase-decarboxylating (Uniprot D8PMS0), transketolase (D8PWF8), and transaldolase (D8PXY7), [73,75]. All identified enzymes were significantly enriched in sample A2. Erythritol is becoming increasingly popular as a natural sweetener, used in low-calorie foods and beverages, and as an additive with artificial sweeteners. Research has focused on reducing production costs through fermentation optimization to meet the rising demand in various industries [73].

Sample A2 showed a higher amount of enzymes involved in the tricarboxylic acid cycle, another relevant pathway closely connected with oxaloacetate biosynthesis. The production of oxalic acid in wood decay fungi is essential during the vegetative phase to derive energy from glucose oxidation [38]. Moreover, the production of oxalic acid prevents the inhibition of ligninolytic enzymes caused by an increase in reactive oxygen species [76]. The main enzymes involved in this pathway are malate synthase (D8Q9D5), oxalate oxidase (D8Q486), and isocitrate lyase (D8Q077), which are expressed at significantly higher levels in sample A2 tan in samples A1 and B.

Finally, the biosynthesis of inositol-phosphate is one of the most complex metabolic pathways related to growth and metabolic adaptation, as well as upregulation during sexual development [77]. Inositol-1-monophosphatase (EC 3.1.3.25) was also identified in the mycelium of *S. commune*, but there were not many differences in its expression among the three extraction methods: A1, A2 and B.

Activation of these metabolic processes leads to the production of reactive oxygen species. The proteomic profile of *S. commune* clearly indicates the expression of various enzymes involved in detoxifying reactive species, such as superoxide dismutases, catalases, and peroxidases [78]. However, it should be noted that in wood-decay mushrooms, peroxidases, in turn, include both enzymes involved in detoxification and lignin degradation, the latter providing self-protection in the fungal organism [22]. In the present study, no lignin was added to the culture medium and peroxidase activity was, therefore, related to basic metabolic functions.

## 5. Conclusions

This study highlights the significance and innovation of utilizing enzymes extracted from a fungal species renowned for its dual roles in medicinal applications and myco-remediation. By conducting the research under standardized growth conditions, we achieved a “general” enzymatic characterization free from the influence of internal or external stressors, thereby capturing the intrinsic metabolic potential of this species. The extraction method A2 proved critical in maximizing the yield and diversity of isolated enzymes, facilitating the recovery of proteins from various cellular compartments, and ensuring a comprehensive proteomic profile. This approach enabled the identification of enzymes with high industrial and nutraceutical and myco-remediation relevance, including trehalose synthase and trehalase, critical for trehalose biosynthesis, a process that is currently complex and costly. These fungal-derived enzymes could streamline the study of trehalose biosynthetic pathways in specific fungal species.

Additionally, this study identified significant levels of peroxidases and catalases, enzymes well-known for their antioxidant properties. Further investigations are needed to determine their potential medical applications, especially in oxidative stress management. Notably, the presence of CAZy enzymes is of particular interest for the dietary use of *S. commune,* as they may positively influence gut flora and enhance intestinal health. With the growing demand for bioactive-enriched products, *S. commune* could be considered a valuable source of proteins and bioactive molecules for novel food production.

In conclusion, given this fungal species’ high enzymatic potential under standard growth conditions and the demonstrated advantages of the A2 extraction method, future research could focus on optimizing the expression of industrially and nutraceutical-relevant enzymes. This research establishes a foundation for utilizing this fungal species as a sustainable source of bioactive enzymes with extensive applications in biotechnology, medicine, and environmental science.

## Figures and Tables

**Table 1:** GO-CC terms statistically enriched in at least one of the samples, A1, A2, and B, when compared to the whole proteome. For each sample, the total number of proteins for each GO-CC is reported in the first column. The *p*-values were calculated in DAVID using Fisher’s Exact test. *p*-values < 0.01 are considered significant of an enrichment (indicated by the symbol *).

	Sample A1		Sample A2		Sample B	
Cellular Component	# Proteins	*p*-Value	# Proteins	*p*-Value	# Proteins	*p*-Value
Cytoplasm	71	8.39 × 10^−12^ *	139	4.03 × 10^−26^ *	99	2.53 × 10^−17^ *
Ribonucleoprotein complex	51	1.15 × 10^−18^ *	59	8.21 × 10^−10^ *	37	8.88 × 10^−4^ *
Ribosome	52	5.46 × 10^−24^ *	56	4.38 × 10^−12^ *	38	1.71 × 10^−6^ *
Mitochondrial inner membrane	12	1.00	31	6.23 × 10^−4^ *	11	1.00
Eukaryotic translation initiation factor 3 complex	5	1.00	11	1.74 × 10^−5^ *	8	9.40 × 10^−3^ *
Eukaryotic 48S preinitiation complex	3	1.00	9	3.73 × 10^−4^ *	7	0.02
Proteasome regulatory particle, base subcomplex	8	4.06 × 10^−5^ *	9	3.73 × 10^−4^ *	7	0.02
Eukaryotic 43S preinitiation complex	3	1.00	9	3.73 × 10^−4^ *	7	0.02
Chaperonin-containing T-complex	0	1.00	8	1.73 × 10^−3^ *	8	8.69 × 10^−5^ *
Proteasome core complex, alpha-subunit complex	7	4.80 × 10^−5^ *	7	7.99 × 10^−3^ *	7	5.85 × 10^−4^ *
Proteasome core complex, beta-subunit complex	6	4.62 × 10^−4^ *	6	0.04	5	0.14
Proteasome complex	6	4.62 × 10^−4^ *	6	0.04	5	0.14

**Table 2:** Number of proteins, quantification (total PSMs), and fold change values (calculated with FUNRICH) for samples A2 and A1, divided per cellular component. The fold change value is the ratio of protein expression based on PSMs. When comparing samples A2 and A1, a fold change value greater than 1.0 indicates a protein overexpressed in A2. CC terms with at least six proteins identified and over 20 PSMs in sample A2 and with a fold value equal to or higher than 1.0 (relative to sample A1) are shown. The cellular components “Cytoplasm” and “Membrane” were assigned to proteins where no more specific cellular component was available.

Cellular Component	# Identified Proteins		Quantification (PSMs)		Fold Change Value
	Sample A2	Sample A1	Sample A2	Sample A1	
Chaperonin-Containing T-Complex	8	0	147.5	0	3280.7
Microtubule	9	3	75.5	2.4	7.0
Arp2/3 Protein Complex	6	3	21.9	1.3	3.7
Eukaryotic 43S Preinitiation Complex	9	3	72	4.3	3.7
Eukaryotic 48S Preinitiation Complex	9	3	72	4.3	3.7
Eukaryotic Translation Initiation Factor 3 Complex	11	5	84.1	5.3	3.5
Golgi Membrane	14	5	85.6	5.6	3.4
Mitochondrial Inner Membrane	31	12	129.2	14.1	2.0
Plasma Membrane	17	6	106.5	12.3	1.9
Proteasome Regulatory Particle, Base Subcomplex	9	8	120.7	15.7	1.7
Cytosol	10	3	42.1	7	1.3
Endoplasmic Reticulum Membrane	24	8	75.1	12.5	1.3
Proteasome Complex	6	6	42	7.3	1.2
Nucleus	81	38	392.6	70	1.2
Spliceosomal Complex	16	7	32.7	6	1.2
Mitochondrion	29	17	207.9	41.3	1.1
Respirasome	9	5	28.7	5.7	1.1
Cytoplasm	139	71	1051.5	210	1.1
Membrane	183	76	601.2	124	1.1

**Table 3:** Number of proteins, quantification (total PSMs), and fold change values (calculated with FUNRICH) for samples A2 and B, divided per cellular component. The fold change value is the ratio of protein expression based on PSMs. When comparing samples A2 and B, a fold change value greater than 1.0 indicates a protein overexpressed in A2. CC terms with at least six proteins identified and over 20 PSMs in sample A2 and with a fold value equal to or higher than 1.0 (relative to sample B) are shown.

Cellular Component	# Identified Proteins		Quantification (PSMs)		Fold Change Value
	Sample A2	Sample B	Sample A2	Sample B	
Respirasome	9	3	28.7	5	3.0
Endoplasmic Reticulum Membrane	24	9	75.1	17.2	2.3
Golgi Membrane	14	9	85.6	20.7	2.2
Ribosome	56	38	449.7	108.9	2.2
Ribonucleoprotein Complex	59	37	438.8	109.6	2.1
Mitochondrial Inner Membrane	31	11	129.2	36.4	1.9
Cellular Anatomical Entity	7	5	53.4	16.2	1.7
Mitochondrion	29	20	207.9	63.2	1.7
Spliceosomal Complex	16	7	32.7	10	1.7
Chaperonin-Containing T-Complex	8	8	147.5	47.7	1.6
Small Ribosomal Subunit	7	6	78.8	25.7	1.6
Cytoplasm	139	99	1051.5	438.6	1.3
Eukaryotic Translation Initiation Factor 3 Complex	11	8	84.1	36.8	1.2
Eukaryotic 43S Preinitiation Complex	9	7	72	31.7	1.2
Eukaryotic 48S Preinitiation Complex	9	7	72	31.7	1.2
Proteasome Regulatory Particle, Base Subcomplex	9	7	120.7	53.7	1.2
Membrane	183	109	601.2	289.8	1.1
Microtubule	9	7	75.5	37	1.1
Plasma Membrane	17	15	106.5	53.4	1.1
Endoplasmic Reticulum	13	9	92	47	1.0

**Table 4:** The enzymes involved in the six molecular pathways investigated are listed in the table, and the difference in expression between the three samples is compared. For each enzyme, the UniProt Accession Number (first column) is indicated, the recommended name, EC number, and alternative name (second column), and the average PSMs for each sample (third, fourth, and fifth columns). The enzymes indicated with an asterisk in the A1 and B columns show a significant decrease with respect to A2 (*t*-test, *p*-values < 0.01 are considered significant). The molecular pathways in which the proteins of interest were identified are indicated in bold. Proteins that were detected at the highest concentration using the A2 extractive method are highlighted in both bold and underlined.

**Key Enzyme**	**Trehalose Biosynthesis**	PSMs Sample A1	PSMs Sample A2	PSMs Sample B
**D8QC44**	Trehalose-6-phosphate synthase (EC 2.4.1.15) (UDP-glucose-glucosephosphate glucosyltransferase)	0.0 *	10.7	2.0 *
D8QIA1	Trehalase (EC 3.2.1.28) (Alpha-trehalose glucohydrolase)	0.0	2.3	0.0
**Q2HZZ3**	Trehalose phosphorylase (EC 2.4.1.231)	13.7 *	42.0	23.0 *
	**Response to oxidative stress**	PSMs Sample A1	PSMs Sample A2	PSMs Sample B
**D8PT51**	Superoxide dismutase (EC 1.15.1.1)	8.3 *	20.0	3.0 *
D8Q7V6	Superoxide dismutase (EC 1.15.1.1)	1.7 *	8.0	0.0
S5VRV3	Superoxide dismutase (EC 1.15.1.1)	0.7	2.3	0.0 *
D8PMW1	Catalase (EC 1.11.1.6)	0.0	2.7	4.3
**D8PTL6**	Catalase (EC 1.11.1.6)	1.3 *	23.0	4.0 *
D8QLS5	Heme peroxidase	0.0	1.3	0.0
**D8QBR0**	Peroxidase (EC 1.11.1.-)	1.0 *	12.7	5.7 *
	**Pentose-phosphate Shunt**	PSMs Sample A1	PSMs Sample A2	PSMs Sample B
**D8PMS0**	6-phosphogluconate dehydrogenase, decarboxylating (EC 1.1.1.44)	15.7 *	35.7	14.3 *
D8PP98	Ribulose-phosphate 3-epimerase (EC 5.1.3.1)	1.7	2.0	2.7
D8PNV4	Glucose-6-phosphate 1-dehydrogenase (EC 1.1.1.49)	0.7 *	15.7	1.7 *
D8PNV6	Glucose-6-phosphate 1-dehydrogenase (EC 1.1.1.49)	1.0	3.7	0.0 *
D8PN33	6-phosphogluconolactonase (6PGL) (EC 3.1.1.31)	1.7 *	5.0	0.0 *
**D8PXY7**	Transaldolase (EC 2.2.1.2)	14.3	22.7	21.7
**D8PWF8**	Transketolase (EC 2.2.1.1)	5.7 *	24.0	4.3 *
	**Tricarboxylic acid cycle**	PSMs Sample A1	PSMs Sample A2	PSMs Sample B
D8QDQ7	Isocitrate dehydrogenase [NADP] (EC 1.1.1.42)	1.7*	5.3	0.0 *
**D8Q9D5**	Malate synthase (EC 2.3.3.9)	9.7 *	33.7	14.7 *
**D8Q486**	Oxalate oxidase	3.3 *	13.3	10.3 *
D8QFS7	Malate dehydrogenase (EC 1.1.1.37)	3.0 *	15.3	21.7 *
D8QLH9	Malate dehydrogenase (EC 1.1.1.37)	3.0 *	15.7	19.3 *
D8Q525	Citrate synthase	6.7 *	22.7	2.0 *
D8PX28	Citrate synthase	0.0 *	8.7	0.7 *
D8QEN1	ATP citrate synthase (EC 2.3.3.8) (ATP-citrate (pro-S-)-lyase) (Citrate cleavage enzyme)	0.7 *	54.7	31.0 *
**D8PMP1**	Isocitrate lyase	1.7	1.7	0.0 *
**D8Q077**	Isocitrate lyase	8.7 *	26.7	13.7 *
D8PZN4	Trehalase (EC 3.2.1.28) (Alpha-trehalose glucohydrolase)	6.7 *	22.7	0.0 *
	**Carbohydrate-Active Enzymes (CAZymes)**			
	**Carbohydrate metabolic processes** **(GHs class)**	PSMs Sample A1	PSMs Sample A2	PSMs Sample B
D8PUK9	Glycoside hydrolase family 31 protein/ beta-glucosidase	2.7 *	12.0	15.7
D8PJY8	Glycoside hydrolase family 125 protein	0.3	4.7	6.7
D8PK12	Glycoside hydrolase family 43 protein	0.0	0.0	1.0
D8PL55	Glycoside hydrolase family 43 and carbohydrate-binding module family 35 protein	1.3	1.3	2.7
D8PLY0	Glycoside hydrolase family 76 protein	0.0	0.3	0.7
D8PMG3	Glycoside hydrolase family 92 protein	1.7	6.0	8.0
D8PMG6	Glycoside hydrolase family 92 protein	0.3	3.0	4.7
D8PMH4	Glycoside hydrolase family 2 protein	1.7	1.7	0.0 *
D8PPV4	Glycoside hydrolase family 92 protein	3.3	0.7	5.7 *
D8PS75	Glycoside hydrolase family 18 protein	1.0 *	5.0	3.3
D8PSJ0	Glycoside hydrolase family 2 protein	0.0	0.0	0.7
D8PVQ7	Glycoside hydrolase family 5 protein/ glucan 1,3- beta-glucosidase	0.7	0.7	1.0
D8PXL2	Glycoside hydrolase family 5 protein/ glucan 1,3- beta-glucosidase	0.3	0.0	0.0
D8PXU7	Glycoside hydrolase family 15 protein	1.7	4.0	0.7 *
D8QJY6	Glycoside hydrolase family 95 protein	0.0	1.0	1.3
D8PJV0	Beta-glucosidase (EC 3.2.1.21)	0.0	1.3	0.0
**D8PS34**	1,4-alpha-glucan-branching enzyme (EC 2.4.1.18) (Glycogen-branching enzyme)	1.3	20.3	4.0
D8PNW5	Beta-glucosidase	0.0	1.3	2.3
D8PQP1	glucan 1,3-beta-glucosidase	0.7 *	3.7	0.0 *
**D8PU51**	Beta-glucosidase	5.3	10.0	22.0 *
**D8PUK9**	Beta-glucosidase	2.7 *	12.0	15.7
**D8Q8Q6**	Beta-glucosidase	0.0	2.0	0.0
**D8Q3Q9**	Beta-galactosidase (EC 3.2.1.23)	1.0	7.3	8.0
**D8PK76**	Beta-galactosidase (EC 3.2.1.23)	2.0	5.7	7.7
**D8PM67**	Beta-galactosidase (EC 3.2.1.23)	0.0	0.3	0.3
**D8Q3Q9**	Beta-galactosidase (EC 3.2.1.23)	1.0	7.3	8.0
**D8PQ92**	Alpha-galactosidase (EC 3.2.1.22) (Melibiase)	3.3 *	12.3	15.7
**D8QGJ4**	Alpha-mannosidase (EC 3.2.1.24)	16.7	32.0	7.3 *
	**Carbohydrate metabolic processes** **(GTs class)**	PSMs Sample A1	PSMs Sample A2	PSMs Sample B
D8Q7P4	Glycosyltransferase family 32 protein	0.0	1.0	0.0
D8QHQ3	Glycosyltransferase family 20 protein	0.3 *	10.7	0.0 *
**D8PXY7**	Transaldolase (EC 2.2.1.2)	14.3	22.7	21.7
	**Inositol phosphatase dephosphorylation**	PSMs Sample A1	PSMs Sample A2	PSMs Sample B
**D8PM90**	Inositol-1-monophosphatase (EC 3.1.3.25)	2.0	1.7	3.0

## Data Availability

All data generated or analyzed during this study are included in this manuscript and its Supplementary Materials. The mass spectrometry proteomics data have been deposited to the ProteomeXchange Consortium via the PRIDE partner repository with the dataset identifier PXD048036 and https://doi.org/10.6019/PXD048036 (accessed on 2 February 2025).

## Supplementary Materials

The following supporting information can be downloaded at https://www.mdpi.com/article/10.3390/jof11020120/s1, Figure S1. SDS-PAGE analysis of S. commune protein extracts obtained using three methods. Lane 1: Molecular weight marker (MWM); Lane 2: Soluble fraction extracted with Method A1 (Tris buffer, mechanical disruption); Lane 3: Insoluble fraction solubilized with Method A2 (urea-based buffer); Lane 4: Protein extract obtained with Method B (TCA precipitation and urea-based solubilization). Equal volumes of each sample were loaded. File S2. Protein list of all identified proteins in the three technical replicates (TR1, TR2, TR3) for the three samples (A1, A2, B). In the table for each protein these attributes are indicated: UniProt Accession, Protein Name, Gene Name, Peptide Spectrum Matches (PSMs) for each technical replicate, Average of PSMs for each sample. File S3. List of enriched Gene Ontology Biological Process (GO-BP) terms considering all identified proteins. For each term in the table are reported: GO-BP term; number of identified proteins annotated in the sample; UniProt Accession of identified proteins; total number of annotated proteins in sample; number of *S. commune* proteins annotated; total number of *S. commune* annotated with GO term; fold enrichment.

## Abbreviations

The following abbreviations are used in this manuscript:

BP	Biological process
CAZymes	Carbohydrate-active enzymes
CC	Cellular Component
GO	Gene Ontology
LC-MS/MS	Liquid Chromatography with tandem mass spectrometry
ME	Malt Extract
MEA	Malt Extract Agar
PSM	Peptide Spectrum Match
TCA	Trichloroacetic acid
