# Supplementary material for: Improving the Proteome-Mining of Schizophyllum commune to Enhance Medicinal Mushroom Applications
Source: J Fungi (Basel). 2025 Feb 5;11(2):120. doi: 10.3390/jof11020120 (PMC11856175; doi:10.3390/jof11020120)
Supplement: Supplementary file 1 [file jof-11-00120-s001.zip › Supplementary Tables Captions.pdf]

Supplementary Tables Captions

Supplementary Table S1 Protein list of all identified proteins in the three technical replicates (TR1, TR2, TR3) for the three samples (A1, A2, B). In the table for each protein these attributes are indicated: UniProt Accession, Protein Name, Gene Name, Peptide Spectrum Matches (PSMs) for each technical replicate, Average of PSMs for each sample.

Supplementary Table S2 List of enriched Gene Ontology Biological Process (GO-BP) terms considering all identified proteins. For each term in the table are reported: GO-BP term; number of identified proteins annotated in the sample; UniProt Accession of identified proteins; total number of annotated proteins in sample; number of *S. commune* proteins annotated; total number of *S. commune* annotated with GO term; fold enrichment.
