# Supplementary material for: Improving the Proteome-Mining of Schizophyllum commune to Enhance Medicinal Mushroom Applications
Source: J Fungi (Basel). 2025 Feb 5;11(2):120. doi: 10.3390/jof11020120 (PMC11856175; doi:10.3390/jof11020120)
Supplement: Supplementary file 1 [file jof-11-00120-s001.zip › Supplementary_Material_S1.pdf]

Supplementary Material S1

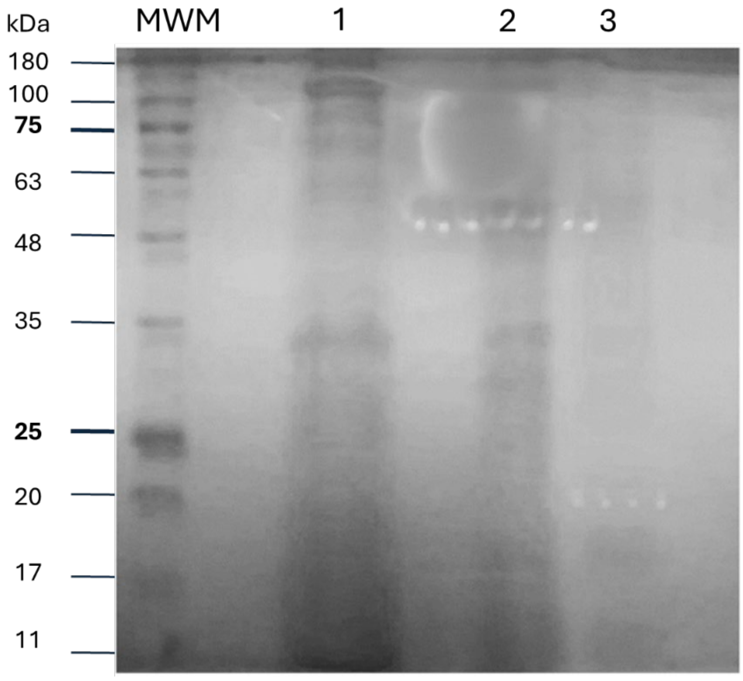

Figure S1. SDS-PAGE analysis of *S. commune* protein extracts obtained using three methods. Lane 1: Molecular weight marker (MWM); Lane 2: Soluble fraction extracted with Method A1 (Tris buffer, mechanical disruption); Lane 3: Insoluble fraction solubilized with Method A2 (urea-based buffer); Lane 4: Protein extract obtained with Method B (TCA precipitation and urea-based solubilization). Equal volumes of each sample were loaded.
